# Theory, evidence and Intervention Mapping to improve behavior nutrition and physical activity interventions

**DOI:** 10.1186/1479-5868-2-2

**Published:** 2005-04-04

**Authors:** Johannes Brug, Anke Oenema, Isabel Ferreira

**Affiliations:** 1 Department of Public Health, Erasmus University Medical Center, PO Box 1738, 3000 DR Rotterdam, The Netherlands

## Abstract

**Background:**

The present paper intends to contribute to the debate on the usefulness and barriers in applying theories in diet and physical activity behavior-change interventions.

**Discussion:**

Since behavior theory is a reflection of the compiled evidence of behavior research, theory is the only foothold we have for the development of behavioral nutrition and physical activity interventions. Application of theory should improve the effectiveness of interventions. However, some of the theories we use lack a strong empirical foundation, and the available theories are not always used in the most effective way. Furthermore, many of the commonly-used theories provide at best information on *what *needs to be changed to promote healthy behavior, but not on *how *changes can be induced. Finally, many theories explain behavioral intentions or motivation rather well, but are less well-suited to explaining or predicting actual behavior or behavior change.

For more effective interventions, behavior *change *theory needs to be further developed in stronger research designs and such change-theory should especially focus on how to promote action rather than mere motivation. Since voluntary behavior change requires motivation, ability as well as the opportunity to change, further development of behavior change theory should incorporate environmental change strategies.

**Conclusion:**

Intervention Mapping may help to further improve the application of theories in nutrition and physical activity behavior change.

## Background

Is there "nothing more practical than a good theory" in improving behavioral nutrition and physical activity interventions? And which theories are indeed good enough to help us improve the practice of encouraging people to adopt healthier diets and physical activity patterns? The International Journal of Behavioral Nutrition and Physical Activity (IJBNPA) recognized the importance of this issue and encouraged a 'theory debate' [1]. Jeffery started the debate by sharing his experiences with and views on applying theories in weight management and weight loss interventions [2]. His conclusion is that we focussed too much on social cognition models and that these models proved to be not very practical. He was not able to find much evidence from his own studies that using such theories improved the effectiveness of interventions. Rothman contributed to the debate by positing that theory should evolve based on rigorous empirical evidence and that intervention research is one of the best ways to evaluate and refine behavior change theory [3]. Rothman further stated that already much attention has been given to explaining how theories should be applied, and that now greater emphasis should be given to further refining or rejecting theoretical principles. In the present contribution to the theory debate, we argue that it is still very necessary to further improve the process that guides which theories are applied in behavior change interventions, how these theories are applied, as well as to further improve and integrate existing theories.

### What is theory and why do we need it?

**Figure 1:**
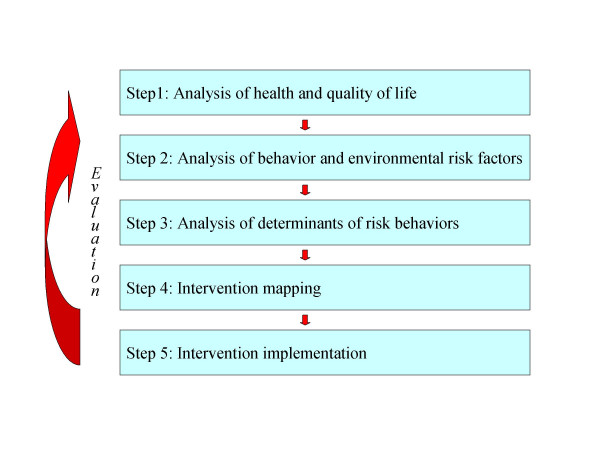
A Model for Planned Health Education and Promotion

Since the publication of Green and Kreuter's Precede and Precede-Proceed models [4], the health behavior promotion area has recognized the importance of careful theory-based intervention planning. According to these, and other similar planning models [5], the first step in health-promotion planning is the identification of health problems that are serious and/or prevalent enough to justify spending time, money and other resources. In the second step, the behavioral risk factors for the health problems need to be identified. Step 3 is to investigate the mediators or determinants of these risk behaviors after which these determinants should be translated into intervention goals, change strategies and methods, that need to be integrated in a comprehensive intervention package (step 4) that can be implemented and disseminated (step 5). Each step should preferably be evidence-based (see Figure 1).

Behavioral theories are mostly used for step 3 of the planning process. Since free choice and autonomy are important values in many societies, and what we eat or how much we exercise are believed to be part of free choice, people choose to a large extend what they eat, and if they are physically active. With very few exceptions (small children, certain groups of institutionalized people), diet and physical activity (PA) behaviors can thus not be influenced directly; instead, we need to influence people's choices. What people choose to eat or do is influenced by a complex, interrelated set of so-called 'mediators' or 'determinants' of nutrition and PA behaviors, including different cognitions as well as environmental factors such as food availability and accessibility [6]. Successful behavior change interventions are dependent on the ability to influence these mediators. Rothschild posited that these mediators can be divided in three broad categories: motivation, abilities and opportunities [7]. Thus, complex combinations of motivations, abilities and opportunities determine diet and PA behaviors. Their relative importance, as well as the underlying beliefs of these determinants, are likely to differ across different populations, as well as between individuals within populations, depending on their personal, social, and environmental circumstances. Furthermore, since these circumstances are liable to change, the most relevant specific determinants may also change over time.

Since the second half of the last century, evidence-based medicine came into fashion. The urgency to base our efforts to improve health or to prolong life on scientific evidence was also transferred to public health and health promotion. In 1998, for example, the World Health Assembly stated that all member states should adopt an evidence-based approach to health promotion [8]. However, what counts as evidence may be debatable. In clinical practice, the randomized controlled trial (RCT) is the 'gold standard' to obtain evidence. An RCT ensures good internal validity, but may lack external validity especially in evaluation of complex behavior-change interventions [8]. The effects of a diet and PA change intervention, based on an inventory of the mediators of change in a specific population and tested among that population, may not be the same in another population with different motivations, abilities or opportunities. Does this mean that evidence on significant mediators of change and effective interventions are relevant only for that specific population under those specific circumstances and thus that it is in fact impossible to build a real evidence-base for behavior change interventions? We don't think so: we should use evidence obtained in specific populations, and under specific circumstances to build, refine and improve behavior change theory.

The Collins Cobuild English Language Dictionary provides two meanings for the word 'theory'. According to the first meaning, a theory is "an idea or set of ideas to explain something. It is based on evidence and careful reasoning, but it cannot be completely proved". The second meaning of a theory according to Collins is "an idea about something that is based on a lot of thinking but not on actual knowledge or evidence".

What we can derive from research, research that includes RCTs but also formative and process evaluation, as well as other forms of impact and effectiveness evaluations [8,9], are evidence-induced general ideas, about which categories of mediators, and which intervention *strategies *and *methodologies*, as well as intervention *channels *may work for influencing certain mediators of behavior change. In other words: we need to build behavior-change theory. These theories should thus be the weighted, systematic, summarized and carefully interpreted results of what has been found in empirical studies directly or indirectly related to nutrition and PA behavior and behavior change. Therefore, we argue that theory-based – in the first aforementioned meaning of the word theory – health behavior interventions is the equivalent of the evidence-based approach in clinical practice. Therefore, theory-based interventions are the only acceptable way to proceed in promotion of healthy diet and PA habits. This, however, implies that we are highly dependent on the quality of the theories and on how these theories are applied in intervention development and implementation. Behavioral nutrition and PA research is a relatively young scientific discipline. Diet and PA behaviors are complex behavioral categories and evidence-induced behavioral nutrition and PA theory is still in its developmental phase and currently too many nutrition and PA behavioral interventions are still based on theory in its second meaning.

In the remainder of this position paper we will, therefore, argue that in healthy nutrition and PA behavior promotion

• we may not always use the available theories in the right way,

• many theories still lack a strong empirical foundation,

• we tend to use theories that are too much focused on the individual and on motivational processes, and

• we may be too inclined to apply a single theory approach.

The Intervention Mapping protocol introduced by Bartholomew and colleagues [10] suggests specific steps that guide problem-driven development, application and integration of nutrition and PA behavior-change theories. IM proposes a systematic way to proceed from knowledge about behavioral determinants to specific change goals, and subsequently to intervention methods and strategies based on the production of intervention matrixes. Such matrices finally develop into an 'intervention map' that make the translation of objectives to change strategies to actual intervention activities explicit [10,16]. In the next discussion paragraph we will therefore refer to approaches suggested in IM that may help to improve the application of behavior theory in intervention development.

## Discussion

### Behavior and behavior change

Theories that are used to inform nutrition and PA behavior-change interventions, are often theories primarily meant to understand, i.e., to explain or predict, behaviors [11]. Applying such theories comes with two important problems. First, a determinant of behavior is not the same as a determinant of behavior *change*. In a study based on the Theory of Planned Behavior [12,13], we found that a majority of Dutch adults had positive attitudes, subjective norms and perceived control to eat a low fat diet, and that these presumed determinants were highly associated with the intention to eat a low fat diet [14]. More than 80% of the Dutch adult population was eating a diet higher in fat than the official Dutch recommendation at that time (fat intake below 35 percent of total energy intake fat). Thus, it appeared that the conditions for encouraging the Dutch population to eat less fat were very positive. However, further research showed that attitudes, norms, perceived control, as well as intentions to *reduce *fat intakes were much less positive [14]. Most people incorrectly thought their diet was already low in fat, and had positive attitudes, perceived norms, control beliefs and intentions to keep eating what they already did [15]. These findings illustrate the need to apply the available theories to try to explain and predict the desired behavior *change *instead of the status quo of healthy or unhealthy behavior, and thus to measure attitudes, subjective norms and perceived control toward behavior change. Intervention Mapping (IM) is a framework for effective theory- and evidence-based decision-making at each step of the process of intervention development, implementation and evaluation of health-promotion interventions [10]. IM argues that for health promotion intervention development we should first translate the health-related behavior (e.g. high fat intake) into a health-promoting behavior or behavior change (e.g. fat reduction) and then search for determinants of the required change, instead of predictors of present behavior.

Second, theories that help us to gain insight into possible determinants of nutrition and PA behaviors or behavior-change, do not directly tell us how to modify these behaviors. Such theories only help us to find out *what *needs to be modified to induce behavior-change [2,3]. We need to use and build better theories that guide us in how the determinants of behavior-change can be modified; how to translate behavior change determinants into behavior-change methods, strategies and actual intervention tools.

### Association or determination?

Attempts have been made to develop behavior-*change *theories. One of the most popular theories used for the development of nutrition and PA behavior-change interventions is the Transtheoretical model and its popular stages of change concept. Stages of change has such a strong appeal because it is brief, has high face validity, and can be easily explained, also to the non-behavioral scientist. Thus, it is readily applicable in intervention development and has been used to develop a range of different nutrition and PA interventions (see [17,18]). Stages of Change is, however, also exemplary for the still rather weak evidence for the theories we use in behavior nutrition and PA research. The evidence for Stages of Change comes almost solely from cross-sectional studies [17,19]. For example, based on cross-sectional associations, pros, cons and self-efficacy are regarded as stage-transition determinants and are used to tailor interventions to each stage of change. However, such cross-sectional associations do not prove that these factors predict, let alone cause stage-transitions or behavior change [19]. Different authors have argued that longitudinal and experimental studies are needed to validate behavior and behavior-change theories better [17,19,20]. In a series of studies that were recently conducted in the Netherlands, the associations found between presumed stage transition determinants and stages of change in cross-sectional studies, could not be replicated in longitudinal analyses [21]. Furthermore, stages of change lacked stability over time, even within a short time interval of only three days [21,22]. As others have also argued, such study results make the validity of one of the most often used behavior change theories rather doubtful [19]. We thus strongly support Rothman's suggestion to use intervention research to test and further refine behavior change theory [3], and Jeffery's experiences show that such rigorous, true experimental tests of theory, can be disappointing [2].

### From motivation to action

Nevertheless, there is evidence that interventions that have applied the stages of change concept are more effective than non-stage matched interventions, at least for short term effects [17]. Perhaps the most important contribution of TTM is the distinction between a motivational phase and a volitional phase in behavior change [23]. The distinction between motivation and action indeed appears to be very relevant. Most theories that are used to inform diet and PA change interventions explain quite well motivation or behavioral intentions, but the explained variance for behavior or behavior change is much lower [25,26]. Intention is an important predictor of behavior. In fact, lack of intention almost certainly results in lack of behavior change. However, a positive intention is no guarantee for behavior change [26], especially not for complex, habitual behaviors like nutrition and PA, that depend very much on personal abilities and environmental opportunities. We need theories to design interventions that help people bridge this intention-behavior gap, i.e. theories that improve people's abilities and opportunities to effectively act on their motivations. Such action-oriented self-regulation models focus specifically on the cognitive mechanisms involved in translating an intention to perform a particular behavior into action. The central tenet of self-regulation models is that through the formation of action goals, pursuing these goals and continuing to pursue these goals in the face of difficulties (i.e. coping with difficulties and frustration) successful transformation of motivation into action and maintenance can be accomplished. Self-regulation models provide various strategies for action initiation and goal pursuit, such as forming implementation intentions [27], goal setting and feedback [28], action planning and building on action self-efficacy and coping self-efficacy [29], self-monitoring and skills training (12). The body of evidence regarding the efficacy and applicability of using these strategies in modifying complex health related behavior is now growing [30,31].

Another possible approach to contribute to bridging the intention – behavior gap is to try to accomplish environmental changes; to increase the actual opportunities for healthy nutrition and PA behaviors and/or to reduce the opportunities for unhealthy behaviors. It has, for example, repeatedly been argued that an environment that offers and encourages plenty of opportunities to eat palatable energy-dense foods and to avoid physical activity may make it extremely difficult for people to act on their positive intentions to prevent weight gain [32-35]. These interventions have mostly tried to apply individual behavioral theories to increase people's awareness, motivation, abilities and confidence to face such an environment. More recently, however, so-called social-ecological theory that highlight the importance of environmental influences, has gained more attention [11]. Once again, this theory mainly identifies what needs to be changed in the environment, rather than how this change can be induced. We still lack systematic evidence and careful reasoning (i.e. theory) driven interventions that can change the environment to offer better opportunities for healthy eating and PA. IM might again offer some direction here [10]. In line with ecological models of health behavior, IM distinguishes between individual and environmental determinants of health behavior and argues that interventions may directly or indirectly address the at-risk individuals. In accomplishing environmental change, the indirect pathway should be used: IM has adopted the approach of Simons-Morton and colleagues [36] and suggests that environmental change is most often eventually the result of changes in behavior of ' decision makers' or 'role actors' at the different levels of the environment: interpersonal, organizational, community or societal. For example parents, school management, local, state and national policy makers all determine part of the nutrition and PA environment of school children. The choices and practices (i.e. behavior) of these decision makers shape the environment to a large extent; it is their health-promotion-facilitating behavior that should induce changes in the environment so that the health-related behavior of the people at risk will change. IM therefore argues that environmental change interventions can best be regarded as behavior change interventions aimed at these decision makers. Consequently, planned environmental change intervention development should first explore the important and changeable mediators of the required behavior change, formulate learning and performance objectives to be accomplished, and identify evidence or theory-based intervention strategies to induce the required behavior change among these decision makers. Environment change is thus translated into behavior change among the agents that have decisional power to modify the environment. This may only be a first step toward bringing about changes at different environmental levels, but it at least opens up a way to systematically think about the important issue of translating our revived attention for and growing insight in the importance of environment as a mediator of nutrition and PA behaviors, into environmental change interventions.

### Problem-driven and theory-driven research

In the last paragraph we argued that the shift in attention to social-ecological models is a much-needed development in nutrition and physical activity behavior-change research. However, what we really need are not studies that highlight the importance of individual factors, social factors *or *physical environmental factors in shaping nutrition and PA behaviors. We need more studies that integrate potential determinants at the environmental *and *the individual levels [11], to study the relative importance of motivation, abilities and opportunities [7] as mediators of nutrition and PA behaviors [37-39]. Without such integrative research, it will remain unclear which causal pathways determine behavior-change and which category of determinants is the preferred point of departure for behavior change interventions. In line with what was suggested by Kok and colleagues for applied social psychology research [40], we can distinguish two general directions in nutrition and PA behavior research: theory-driven and problem- or action-driven research [41]. Theory-driven research is conducted to test or improve the validity or applicability of a specific theory of nutrition or PA behavior (see for example [42-45]. Problem-driven or action-driven research is conducted to tackle a specific problem, to explain this problem to the fullest extent and to give direction to possible solutions. Theories are of most importance in problem-driven research, but the main focus is not on testing a theory, but on using insights from different relevant theories in order to solve a problem. Therefore, in such problem-driven research often concepts derived from different theories are used instead of the single-theory perspective of most theory-driven studies. IM has adopted this integrative (i.e. multi-level) problem-driven approach to explore mediators of behavior change and to identify potential behavior change strategies [10,16].

## Conclusion

Theory is the only foothold we have in development of behavior nutrition and physical activity interventions since theories are (or should be) a generalized and careful interpreted systematic summary of empirical evidence. Thus, application of theory should improve the likelihood of effectiveness of interventions. However, most of the theories that are applied in behavior nutrition and PA interventions provide information on *what *needs to be changed to promote healthy behavior but not on *how *change can be induced. Furthermore, some of these theories lack a strong empirical foundation and do better in explaining behavior intentions or motivation than actual behavior or behavior change.

For more effective interventions, behavior *change *theory needs to be further developed with stronger research designs and such change theory should especially focus on how to promote action rather than mere motivation. Since voluntary behavior change requires motivation, ability as well as the opportunity to change, further development of behavior change theory should incorporate environmental change strategies. Intervention Mapping may provide a number of tools to further improve the development and application of theories in interventions to promote nutrition and PA behavior change.

## Competing interests

The author(s) declare that they have no competing interests.

## Authors' contributions

JB initiated this paper and wrote the first draft.

AO and IF discussed the draft paper with JB and provided written comments. AO wrote the paragraph on action-oriented theory.
